# Exercise Barriers in Older Individuals with Alzheimer’s Disease: A Cross-Sectional Study

**DOI:** 10.3390/medicina60091510

**Published:** 2024-09-16

**Authors:** İsmail Uysal, Fatih Özden, Mehmet Özkeskin, Zehra Benzer, Emir İbrahim Işık

**Affiliations:** 1 Department of Health Care Services, Fethiye Vocational School of Health Services, Muğla Sıtkı Koçman University, 48200 Muğla, Turkey; 2 Department of Health Care Services, Köyceğiz Vocational School of Health Services, Muğla Sıtkı Koçman University, 48800 Muğla, Turkey; 3 Department of Physiotherapy and Rehabilitation, Faculty of Health Sciences, Ege University, 35520 İzmir, Turkey; mehmet.ozkeskin@gmail.com; 4 Department of Physiotherapy and Rehabilitation, Institute of Health Sciences, Ege University, 35520 İzmir, Turkey; zehrabnzr@gmail.com; 5 Department of Physiotherapy and Rehabilitation, Faculty of Health Sciences, Çukurova University, 01330 Adana, Turkey; eisik@cu.edu.tr

**Keywords:** cognitive disorders, exercise habituation, physical, psychological

## Abstract

*Background and Objectives*: Defining the exercise habits of individuals with Alzheimer’s Disease (AD) may help to determine optimal rehabilitation programs. This study aimed to investigate the physical and psychological parameters associated with exercise barriers in older individuals with AD, with the goal of informing more effective rehabilitation programs. *Materials and Methods*: A cross-sectional prospective study was conducted with 50 individuals with AD. The individuals were evaluated with the Exercise Benefit/Barriers Scale (EBBS), the Mini-Mental State Examination (MMSE), the Five Times Sit to Stand Test (FTSTS), the Barthel Index (BI), the Tampa Scale for Kinesiophobia (TSK), and the Hospital Anxiety and Depression Scale (HADS). *Results*: There was a significant positive correlation between age with EBBS-Exercise Barriers (r = 0.308; *p* = 0.029) and EBBS-Total Score (r = 0.295; *p* = 0.038). There were significant negative correlations between the time of diagnosis with EBBS-Exercise Benefits (r = −0.569; *p* = 0.000), EBBS-Exercise Barriers (r = −0.324; *p* = 0.022), and EBBS-Total Score (r = −0.508; *p* = 0.000). There was a positive correlation between MMSE and EBBS-Exercise Benefits (r = 0.465; *p* = 0.001), EBBS-Exercise Barriers (r = 0.471; *p* = 0.001) and EBBS-Total Score (r = 0.519; *p* = 0.000). There were significant positive correlations between FTSTS and EBBS-Exercise Barriers (r = 0.340; *p* = 0.016), and EBBS-Total Score (r = 0.280; *p* = 0.049). There were positive correlations between BI and EBBS-Exercise Benefits (r = 0.362; *p* = 0.010), EBBS-Exercise Barriers (r = 0.377; *p* = 0.007), and EBBS-Total Score (r = 0.405; *p* = 0.004). *Conclusions*: Exercise barriers/benefits were associated with cognition and post-diagnosis duration in individuals with AD. Individuals with lower physical function had lower exercise perception. In addition, living with relatives or caregivers led to better exercise benefit scores.

## 1. Introduction

Alzheimer’s Disease (AD) is characterized by the accumulation of β-amyloid (Aβ) plaques outside the cell and neurofibrillary tangles containing tau inside the cell [1]. AD leads to impairment in behavioral and cognitive functions, affecting the individual’s independence in daily life activities. Along with cognitive deficits, decreases in walking and balance ability, which are among the most basic activities of daily living, have been reported in AD [2].

Studies evaluating gait and balance in individuals with AD showed slower walking speeds, lower cadence, and shorter stride lengths in individuals with AD [3,4]. These differences in gait and balance cause individuals with AD to experience more falls than do healthy older people. This issue has been associated with fear of falling and restriction in activities of daily living [5]. Individuals who restrict their activities become physically immobilized and may experience psychological disorders such as depression due to their reduced interaction with the social environment [6].

Another psychological symptom caused by being physically inactive is kinesiophobia. Kinesiophobia is defined as an excessive, irrational, and debilitating fear of movement due to pain or previous injury [7]. Although the existing researches are limited to kinesiophobia in individuals with AD, it is essential to evaluate this, since fear of movement occurs in cognitively and psychologically affected individuals [8].

Several psychological symptoms, such as depression, anxiety, and psychosis, are frequently observed in AD [9,10]. These symptoms have consequences such as loss of functional independence and decreased quality of life [9]. Early treatment of neuropsychiatric symptoms is necessary, both to ensure that the individual maintains independence for a long time and to reduce the burden on the caregiver. Considering the fatal side effects of antipsychotic drugs used in treatment, clinicians have been encouraged to use non-pharmacological treatment methods [11].

Non-pharmacological approaches are used as a complementary option to pharmacological treatments in patients with Alzheimer’s disease [12]. These include physical activities, social activities, music therapy, and animal-assisted therapies [13,14,15,16]. Moreover, the beneficial effects of physical activity as an intervention therapy are supported by numerous systematic reviews and meta-analyses [13]. Exercise therapy is one of the non-pharmacological physical activity-based treatment methods used to treat physical and psychological symptoms in individuals with AD, as it is the cheapest and safest treatment method without side effects. Physical activity and exercise are used to both prevent and treat psychiatric symptoms. Exercise therapy is also essential in treating AD due to its positive effects on increasing blood flow to the brain and improving general health [17].

While the importance of exercise and activity in individuals with AD is recognized, the benefits and barriers of exercise, an indicator of efficiency in therapy, have not been sufficiently studied. Defining the exercise habits of individuals with AD may contribute to the determination of optimal rehabilitation programs in a pragmatic way. Therefore, this study aimed to investigate the physical and psychological parameters associated with exercise barriers in older individuals with AD.

## 2. Materials and Methods

### 2.1. The Subjects and Clinical Setting

A cross-sectional prospective study was conducted at the Alzheimer’s Association in Fethiye and İzmir. A total of 50 individuals diagnosed with AD by a specialist physician participated in the study. The inclusion criteria of the study were: (1) AD diagnosis according to the criteria of the National Institute of Neurological and Communicative Disorders and Stroke and Alzheimer’s Disease, (2) age older than 65 years, (3) ability to communicate and understand verbal and written comments. The study’s exclusion criteria were: (1) comorbid diseases potentially affecting evaluation, (2) refusal to participate, (3) a MMSE of lower than 13 (severe cognitive disorder), (4) presence of early-onset AD (because of their significantly different clinical and neurobiological features), (5) inability to respond to outcome measures. Medication use for the individuals in the study was arranged by the specialist physician.

Permission was obtained from the relatives or legal guardians of the individuals with AD. The study protocol was approved by the ethics committee of Muğla Sıtkı Koçman University (No: 230096/97, Date: 10 October 2023).

### 2.2. Sample Size

Cohen’s d coefficient was considered for the two-tailed hypothesis (effect size = 0.5, an error probability of 0.05, and statistical power of 0.95). Finally, the calculation results showed that at least 42 cases were eligible.

### 2.3. Outcome Measures

Data collection was conducted with face-to-face meetings. An assessment form includes individual and demographic characteristics (e.g., age, gender, body mass index, education, marital status, duration of diagnosis). The individuals were evaluated with the Mini-Mental State Examination (MMSE), the Five Times Sit to Stand Test (FTSTS), the Barthel Index (BI), the Exercise Benefit/Barriers Scale (EBBS), the Tampa Scale for Kinesiophobia (TSK), and the Hospital Anxiety and Depression Scale (HADS).

#### 2.3.1. Mini-Mental State Examination (MMSE)

Developed in 1975 by Folstein and colleagues, this short screening test is the most commonly used test for dementia screening. It consists of 11 questions and is scored out of 30 points. A score of 24 to 30 points is average, 18 to 23 points is mild dementia, and 17 points and below is compatible with severe dementia. It tests orientation, memory, attention, calculation, recall, language, motor function and perception, and visuospatial abilities; its most significant advantage is that it is easy and quick to administer [18].

#### 2.3.2. Five Times Sit to Stand Test (FTSTS)

The FTST is designed to measure individuals’ physical performance. A person sits in a standard chair and then stands, repeating this five times. The time elapsed for these tasks is calculated for the final test results. A lower duration indicates better physical performance outcomes. This test also shows lower extremity muscle strength in older adults, demonstrating the reliability and validity of the FTST in older adults [19].

#### 2.3.3. Barthel Index (BI)

The Barthel index (BI) is a frequently used scale that assesses basic activities related to self-care and mobility. The total score of the BI is 100. The higher the score, the more independent the individual is considered to be [20].

#### 2.3.4. Exercise Benefit/Barriers Scale (EBBS)

EBSS was developed by Sechrist et al. to determine people’s perceptions of the benefits and barriers of exercise. The scale consists of 43 questions, which are scored between 43 and 172. It has two sub-dimensions: Exercise Benefit Scale and Exercise Barrier Scale. Each sub-dimension can be evaluated separately. The total score on the benefit scale is between 29 and 116, and the total score on the barrier scale is between 14 and 56. A high total score on the Exercise Benefit Scale indicates that the individual perceives exercise benefits well. A high total score on the Exercise Barrier Scale indicates that the individual perceives many exercise barriers [21].

#### 2.3.5. Tampa Scale for Kinesiophobia (TSK)

TSK was developed, but not published, by Miller, Kori, and Todd in 1991; it was published by Vlaeyen et al. in 1995 [22]. The TSK, whose Turkish validity and reliability studies were constructed by Yılmaz et al. in 2011 [23]. The average score ranges between 17 and 68. Patients participating in the study were asked to rate each item on a 4-point Likert scale ranging from 1 (strongly disagree) to 4 (strongly agree). The scale measures two constructs labeled somatic focus (5 items) and activity avoidance (8 items) [23].

#### 2.3.6. Hospital Anxiety and Depression Scale (HADS)

The Hospital Anxiety and Depression Scale is a self-administered scale consisting of 14 items split across anxiety and depression subscales, each with a four-point ordinal response format. A recent meta-analysis of diagnostic test accuracy studies reported that using a score of 8 or more as a cut-off point yielded 82% sensitivity and 74% specificity for detecting major depressive disorder; the anxiety scale yielded 78% sensitivity and 74% specificity for detecting generalized anxiety disorder [24].

### 2.4. Statistical Analysis

The analysis of the research data was carried out using the statistical program SPSS 29.0. Descriptive results were given in terms of number, percentage, minimum/maximum, mean, standard deviation, and median. The normal distribution of the variables was analyzed using the Shapiro–Wilk test. Normal distribution was also examined on a group basis in the analysis of differences. When comparing two independent groups, the *t*-test was used when the data showed a normal distribution, the Mann–Whitney U-test was used when the data did not show a normal distribution, and the Kruskal-Wallis’s test was used when comparing three or more independent groups because the data did not show a normal distribution. Bonferroni-corrected multiple comparison tests were used to identify groups with differences when significant differences were found in the Kruskal–Wallis tests. When assessing relationships between continuous variables, the Pearson correlation test was used when the data showed a normal distribution, and the Spearman correlation test was used when the data did not show a normal distribution. A value of *p* < 0.05 was considered statistically significant in the analyses.

## 3. Results

The mean age of the individuals was 75.6 ± 6.2 years. Mean and frequency data on individual and demographic characteristics of the participants are presented in Table 1.

Mean values for clinical evaluations are given in Table 2. The results of the difference analysis, performed to determine whether there is a difference in the evaluation parameters according to the individual characteristics of the individuals, are presented in Table 3. According to these findings, a statistically significant difference was found in the scores of “EBBS-Exercise Benefits”, “EBBS-Exercise Barriers”, and “EBBS-Total Score”, according to the living conditions of the patients (*p* < 0.05). As a result of Bonferonni corrected multiple comparison tests performed to determine between which groups the differences were, it was seen that the mean score for patients living with relatives was higher than the mean of patients living alone in “EBBS-Exercise Benefits” and “EBBS-Total Score”, and the mean of patients living with caregivers was higher than the mean of patients living alone in “EBBS-Exercise Barriers”.

The findings of the correlation analysis to determine the relationships between age, BMI, and time of diagnosis, and clinical evaluation results are presented in Table 4. According to these findings, there was a significant positive correlation between age and MMSE (r = 0.385; *p* = 0.006), FTSTS (r = 0.342; *p* = 0.015), BI (r = 0.293; *p* = 0.039), EBBS-Exercise Barriers (r = 0.308; *p* = 0.029) and EBBS-Total Score (r = 0.295; *p* = 0.038). A significant positive correlation was found between BMI and HADS-Depression (r = 0.346; *p* = 0.014) and a significant negative correlation between BMI and BI (r = −0.335; *p* = 0.017). There were significant negative correlations between the time of diagnosis and MMSE (r = −0.419; *p* = 0.002), EBBS-Exercise Benefits (r = −0.569; *p* = 0.000), EBBS-Exercise Barriers (r = −0.324; *p* = 0.022) and EBBS-Total Score (r = −0.508; *p* = 0.000).

The correlation analysis results between the research scales are presented in Table 5. According to these findings, there was a positive correlation between MMSE and EBBS-Exercise Benefits (r = 0.465; *p* = 0.001), EBBS-Exercise Barriers (r = 0.471; *p* = 0.001), and EBBS-Total Score (r = 0.519; *p* = 0.000). There were significant positive correlations between FTSTS and EBBS-Exercise Barriers (r = 0.340; *p* = 0.016), and EBBS-Total Score (r = 0.280; *p* = 0.049). There were positive correlations between BI and EBBS-Exercise Benefits (r = 0.362; *p* = 0.010), EBBS-Exercise Barriers (r = 0.377; *p* = 0.007), and EBBS-Total Score (r = 0.405; *p* = 0.004).

## 4. Discussion

The present study aimed to investigate the physical and psychological parameters associated with exercise barriers in older individuals with AD. In this cross-sectional study, exercise-related barriers and benefits in individuals with AD were found to be strongly associated with individuals’ cognitive status and time since diagnosis. This important finding suggests that the benefits of and barriers to exercise vary depending on the progression of the disease and cognitive function. In addition, individuals with a lower physical function had lower exercise perception. Besides, living with relatives or caregivers had better exercise and barrier scores.

Physical, psychological, and behavioral parameters—along with cognitive changes—are widely changed in individuals with AD. Exercise therapy is used in AD to improve physical and cognitive functions, reduce neuropsychiatric symptoms, and maintain independence in activities of daily living [25]. However, in these individuals, the perception of exercise may have changed due to cognitive impairment, decreased awareness, and fear of movement.

One of our primary results revealed that individuals with higher cognitive status had a better perception of the benefits of exercise. There are a number of perceived benefits of exercise in individuals with AD that are not limited to health outcomes [26]. Similar to the findings of this study, Karuncharernpanit et al. (2016) reported that individuals with higher cognitive status have a better perception of exercise benefits. The same study reported that families, caregivers, and health professionals of individuals with AD may have positive contributions to the perception of exercise benefits [27]. This positive perception of exercise in the AD population may encourage individuals to engage in physical activity, and thus, physically active individuals may maintain their independence for longer. Therefore, we concluded that individuals with good cognitive status had a higher degree of autonomy in activities of daily living. This result’s primary explanation and inference may be due to the requirement of a great deal of cognitive ability during daily living activities, including financial affairs, shopping, dressing, and bathing, which also depend on cognition, as stated by Njegovan et al. [28]. Since the progression of the disease stage would increase the severity of cognitive impairment, the loss of independence in daily living activities may increase in these individuals gradually. In order to reduce the loss of independence and increase exercise participation, encouragement from health professionals, caregivers, and family can be an incentive to improve the perception of exercise benefits.

The ratio of perceived exercise barriers to perceived benefits determines a person’s health and physical activity behavior. The most significant barriers to physical activity are pain, weakness, lack of motivation, and lack of time [29]. In our study, as a result of the correlation analyses we performed to determine exercise barriers in individuals with AD, we found significant relationships between age, the severity of cognitive impairment, physical performance, and independence in activities of daily living. Aging, severe cognitive impairment, poor physical performance, and high independence in activities of daily living increase the perceived barriers to exercise. Schutzer et al. stated that physical activity decreases with aging. This study also mentioned that older adults avoid exercise due to being weak, frail, and having more health problems [30]. In our population, AD leads to the development of a barrier attitude toward exercise, in addition to aging. People with good cognitive function do physical activity more efficiently and can better maintain this behavior [31]. Individuals with more cognitive impairment have more barrier attitudes towards exercise because of the difficulties they experience in perceiving, implementing, and maintaining exercises. On the other hand, it is known that support programs and cognitive exercise approaches are useful in improving motivational variables that support the maintenance of healthy lifestyle activities such as physical activity, and increase participation in exercise [32,33]. Therefore, it may be beneficial to support physical exercise with support programs and cognitive exercises.

Individuals with AD lose their ability to perform complex activities of daily living in the later stages of the disease, due to cognitive impairments [34]. People who become dependent on a caregiver over time are affected physically and psychologically [35]. Loss of independence restricts individuals with AD and leads to activity avoidance behavior, and the deterioration of physical performance as a result of this situation triggers fear of movement. The findings of our study support this idea in terms of correlational results. Kinesiophobia and anxiety scores were found to be higher in individuals with AD who had less independence in activities of daily living.

Individuals’ living conditions affect their attitudes towards exercise. Our study found that individuals living with relatives had a higher perception of benefits from exercise than individuals living alone. This result supports the finding that having at least one personal supporter is associated with higher exercise participation, as Gauvin et al. stated [36]. Home-based exercise programs with regular support from caregivers are key elements that facilitate adherence to the program in people with AD [37]. In addition, support based on behavior management techniques applied by caregivers improves exercise participation, exercise duration, and long-term effects in people with AD [38]. We consider that individuals with AD living alone have different perceptions towards exercise because they do not receive adequate physical and motivational support. Health professionals, as well as family and caregivers, have an impact on improving exercise perception. Therefore, it is estimated that health professionals’ counseling to encourage exercise may be beneficial for individuals with AD living alone.

Compliance with treatment in physiotherapy is possible with regular attendance of sessions, considering the physiotherapist’s recommendations, and applying the exercise program prepared individually correctly and at the recommended frequency [39]. Determining the factors affecting treatment adherence is essential for physiotherapists to identify patients who are likely to be non-compliant and to ensure the continuity of these patients to the treatment program. In this context, exercise barriers affect patients’ adherence to treatment, and knowing exercise barriers guides physiotherapists in selecting physical activity and exercise programs. Physiotherapists’ awareness of barriers to physical activity and exercise helps them prepare a treatment program tailored to the patient’s needs and to maintain this program in cooperation with the patient [40].

Vseteckova et al. showed that cognitive impairment, anxiety, depression, and decreased activities of daily living were barriers to exercise in their study on older adults with dementia living in nursing homes. Factors such as improvements in physical health, psychological well-being, enjoyment of exercise, increased feelings of self-efficacy and self-worth, and regaining control were identified as facilitators [41]. The findings of our study are in line with this information. Cognitive status, independence in activities of daily living, and physical performance affect individuals’ attitudes toward exercise. However, unlike Vseteckova et al., we did not find a significant relationship between anxiety and depression and exercise barriers in our study. This outcome may be due to the small number of participants included in the study.

Our study may have limitations, such as using a small sample size and not including healthy older people as a control group. The limited sample size may have reduced the statistical power of our study and potentially limited the ability to detect significant differences or trends that may exist in a larger population. This limitation may underestimate the generalizability of our findings, especially across diverse populations. In future studies, increasing the sample size will help confirm the robustness of the observed trends and provide a clearer understanding of the relationship between the variables examined. Although our sample size is comparable to that generally considered in similar studies on older adults, our results need to be repeated in larger samples and with other tasks [42]. A control group consisting of healthy elderly people could not be formed due to the nature of the study, as well as the difficulty of matching to minimize bias and confounding [43]. The lack of a control group limits the ability to draw strong causal conclusions about the effects of the intervention, because we cannot definitively rule out the influence of external factors or the natural progression of the condition. A control group in future studies would allow for more rigorous comparisons and better validation of specific effects of the intervention. On the other hand, basing exercise barriers and benefits on objective data with a standardized measurement based on patients’ current exercise practice levels and capacities may provide more effective results. In addition, since the individuals in our study had cognitive impairments, there may be incompatibility in the answers given in the patient-report scales we used. This may have affected our results. In future studies, the development of short and clear patient reporting scales developed for individuals with AD and their carers will eliminate the possible effect of self-report bias.

## 5. Conclusions

Exercise barriers and benefits were associated with cognition and post-diagnosis duration in individuals with AD. In addition, individuals with lower physical function had lower exercise perception. Further, living with relatives or caregivers led to better exercise and barrier scores.

## Figures and Tables

**Table 1:** Demographic characteristics of the participants.

*n*: 50		Total
Age (years, mean ± SD)		75.6 ± 6.2
Gender (*n*, %)	Women	35 (70.0)
	Men	15 (30.0)
BMI (kg/m^2^, mean ± SD)		25.9 ± 3.3
Living condition (*n*, %)	Partner	27 (54.0)
	Alone	7 (14.0)
	Relatives	14 (28.0)
	Caregiver	2 (4.0)
Marital status (*n*, %)	Married	27 (54.0)
	Single	23 (46.0)
Education (*n*, %)	None	1 (2.0)
	Primary school	24 (48.0)
	Secondary school	8 (16.0)
	High school	13 (26.0)
	University or higher	4 (8.0)
Duration after diagnosis (years, mean ± SD)		5.3 ± 2.9

*n*: number of participants, SD: Standard Deviation, BMI: Body Mass Index.

**Table 2:** The mean scores of the clinical outcomes.

*n*: 50	Minimum	Maximum	Mean	Standard Deviation	Median
MMSE	10.00	25.00	15.90	2.823	15.00
FTSTS	10.12	21.89	14.56	2.389	14.34
BI	65.00	100.00	88.00	10.546	90.00
TSK	33.00	57.00	43.41	4.707	43.00
HADS-Depression	0.00	15.00	7.10	3.688	8.00
HADS-Anxiety	0.00	12.00	4.90	3.436	4.00
EBBS-Exercise Benefits	32.00	98.00	71.72	16.176	72.50
EBBS-Exercise Barriers	20.00	66.00	35.94	9.065	36.00
EBBS-Total Score	54.00	145.00	107.82	22.014	108.50

*n*: number of participants, MMSE: Mini-Mental State Examination, FTSTS: Five Times Sit to Stand Test, BI: Barthel Index, EBBS: Exercise Benefit/Barriers Scale, TSK: Tampa Scale for Kinesiophobia, HADS: Hospital Anxiety and Depression Scale.

**Table 3:** The significance tests and their post hoc analysis according to demographic factors.

	MMSE	FTSTS	BI	TSK	HADS-Depression	HADS-Anxiety	EBBS-Exercise Benefit	EBBS-Exercise Barriers	EBBS-Total Score
**Gender**									
Women	16.03 ± 2.706	14.32 ± 2.167	89.43 ± 11.033	42.89 ± 5.063	6.91 ± 3.534	4.74 ± 3.346	73.31 ± 14.992	35.97 ± 7.998	109.29 ± 21.879
Men	15.60 ± 3.158	15.13 ± 2.841	84.67 ± 8.756	44.65 ± 3.598	7.53 ± 4.121	5.27 ± 3.731	68.00 ± 18.678	35.87 ± 11.501	104.40 ± 22.709
T/Z	−0.374	−1.111 ^T^	−1.818	−1.774	−0.426	−0.501	1.066 ^T^	0.037 ^T^	0.716 ^T^
*p*	0.709	0.272	0.069	0.076	0.670	0.616	0.292	0.971	0.478
**Living Condition**									
Partner ^1^	15.52 ± 2.709	14.48 ± 2.594	87.04 ± 10.120	43.52 ± 4.847	6.30 ± 3.881	4.52 ± 3.857	69.74 ± 17.726	35.56 ± 10.162	105.30 ± 23.070
Alone ^2^	15.86 ± 3.078	14.20 ± 1.620	84.29 ± 13.363	44.39 ± 3.149	8.14 ± 3.388	5.29 ± 2.563	61.43 ± 10.581	30.00 ± 5.802	92.57 ± 15.043
Relatives ^3^	16.21 ± 2.914	15.17 ± 2.194	90.00 ± 9.806	42.71 ± 3.730	7.93 ± 3.583	5.14 ± 2.852	78.79 ± 12.503	38.29 ± 6.888	117.07 ± 18.628
Caregiver ^4^	19.00 ± 2.828	12.66 ± 3.592	100.00 ± 0.000	43.5 ± 14.849	8.50 ± 0.707	7.00 ± 5.657	85.00 ± 7.071	45.50 ± 3.536	130.50 ± 3.536
F/X^2^	2.950	1.946	4.711	1.168	3.075	1.876	8.685	8.297	9.532
*p*	0.399	0.584	0.194	0.761	0.380	0.599	0.034	0.040	0.023
Difference	-	-	-	-	-	-	2 < 3	2 < 4	2 < 3
**Marital Status**									
Married	15.52 ± 2.709	14.48 ± 2.594	87.04 ± 10.120	43.52 ± 4.847	6.30 ± 3.881	4.52 ± 3.857	69.74 ± 17.726	35.56 ± 10.162	105.30 ± 23.070
Single	16.35 ± 2.948	14.66 ± 2.178	89.13 ± 11.145	43.29 ± 4.642	8.04 ± 3.282	5.35 ± 2.886	74.04 ± 14.179	36.39 ± 7.785	110.78 ± 20.817
T/Z	−0.854	−0.260 ^T^	−0.871	−0.439	−1.742	−1.275	−0.936 ^T^	−0.322 ^T^	−0.876 ^T^
*p*	0.393	0.796	0.384	0.660	0.082	0.202	0.354	0.749	0.385
**Education**									
Secondary or lower	15.88 ± 3.244	14.58 ± 2.124	85.40 ± 10.89	43.96 ± 5.358	7.76 ± 3.767	5.60 ± 3.536	72.40 ± 11.136	35.12 ± 7.055	107.52 ± 16.432
High or higher	15.92 ± 2.397	14.54 ± 2.673	90.60 ± 9.717	42.87 ± 3.988	6.44 ± 3.560	4.20 ± 3.253	71.04 ± 20.231	36.76 ± 10.798	108.12 ± 26.818
T/Z	−0.489	0.062 ^T^	−1.716	0.816 ^T^	1.273 ^T^	−1.320	−0.214	−0.636 ^T^	−0.095 ^T^
*p*	0.625	0.951	0.086	0.419	0.209	0.187	0.831	0.528	0.924

T = *t* test; Z = Mann–Whitney U test; X^2^ = Kruskal–Wallis test, MMSE: Mini-Mental State Examination, FTSTS: Five Times Sit to Stand Test, BI: Barthel Index, EBBS: Exercise Benefit/Barriers Scale, TSK: Tampa Scale for Kinesiophobia, HADS: Hospital Anxiety and Depression Scale, ^1^ = partner, ^2^ = alone, ^3^ = relatives, ^4^ = caregiver, bold values indicates significant *p* scores.

**Table 4:** Correlation between patient characteristics and clinical measures.

*n*: 50		Age	BMI	Duration
MMSE	r	0.385	−0.034	−0.419
	*p*	0.006	0.813	0.002
FTSTS	r	0.342 ^1^	0.007 ^1^	−0.052
	*p*	0.015	0.963	0.718
BI	r	0.293	−0.335	−0.255
	*p*	0.039	0.017	0.074
TSK	r	−0.003	−0.023	0.053
	*p*	0.985	0.876	0.714
HADS-Depression	r	0.022 ^1^	0.346 ^1^	0.054
	*p*	0.880	0.014	0.711
HADS-Anxiety	r	−0.266	0.055	0.142
	*p*	0.062	0.706	0.326
EBBS-Exercise Benefits	r	0.239 ^1^	−0.127 ^1^	−0.569
	*p*	0.095	0.379	0.000
EBBS-Exercise Barriers	r	0.308 ^1^	−0.130 ^1^	−0.324
	*p*	0.029	0.367	0.022
EBBS-Total Score	r	0.295 ^1^	−0.142 ^1^	−0.508
	*p*	0.038	0.326	0.000

^1^ Pearson’s correlation test, MMSE: Mini-Mental State Examination, FTSTS: Five Times Sit to Stand Test, BI: Barthel Index, EBBS: Exercise Benefit/Barriers Scale, TSK: Tampa Scale for Kinesiophobia, HADS: Hospital Anxiety and Depression Scale.

**Table 5:** The correlation results between exercise barriers/benefits and other measures.

*n*: 50		MMSE	FTSTS	BI	TSK	HADS-D	HADS-A
EBBS-Exercise Benefits	r	0.465	0.199 ^1^	0.362	−0.176	0.039 ^1^	−0.184
	*p*	0.001	0.165	0.010	0.221	0.789	0.201
EBBS-Exercise Barriers	r	0.471	0.340 ^1^	0.377	−0.153	−0.085 ^1^	−0.210
	*p*	0.001	0.016	0.007	0.290	0.559	0.143
EBBS-Total Score	r	0.519	0.280 ^1^	0.405	−0.160	0.003 ^1^	−0.212
	*p*	0.000	0.049	0.004	0.266	0.981	0.139

^1^ Pearson’s correlation test, MMSE: Mini-Mental State Examination, FTSTS: Five Times Sit to Stand Test, BI: Barthel Index, EBBS: Exercise Benefit/Barriers Scale, TSK: Tampa Scale for Kinesiophobia, HADS: Hospital Anxiety and Depression Scale.

## Data Availability

Dataset available on request from the authors.
